# Membrane interaction of cyanobacterial and chloroplast ESCRT‐III proteins

**DOI:** 10.1111/tpj.70843

**Published:** 2026-04-06

**Authors:** Mirka Kutzner, Benedikt Junglas, Mayank Sharma, Carsten Sachse, Dirk Schneider

**Affiliations:** ^1^ Department of Chemistry, Biochemistry Johannes Gutenberg University Mainz Mainz Germany; ^2^ Ernst‐Ruska Centre for Microscopy and Spectroscopy with Electrons, ER‐C‐3: Structural Biology Forschungszentrum Jülich Jülich Germany; ^3^ Department of Biology Institute of Molecular Plant Biology, ETH Zurich Zurich Switzerland; ^4^ Department of Biology Heinrich Heine University Düsseldorf Düsseldorf Germany; ^5^ Institute of Molecular Physiology Johannes Gutenberg University Mainz Mainz Germany

**Keywords:** ESCRT‐III, IM30, Vipp1, PspA, chloroplast, cyanobacteria, thylakoid membrane, membrane dynamics, membrane remodeling

## Abstract

More than three decades ago, the *inner membrane‐associated protein of 30 kDa* (IM30), also known as Vipp1, was identified in pea chloroplasts to bind to the chloroplast inner envelope membrane. IM30/Vipp1 is a membrane‐associated and soluble stromal protein and is proposed to mediate vesicle formation. Furthermore, it is linked to membrane stabilization processes, as also discussed for its bacterial homolog PspA. More recently, the structures of cyanobacterial IM30/Vipp1 and PspA proteins were resolved, revealing that these proteins belong to the *endosomal sorting complex required for transport III* (ESCRT‐III) superfamily. ESCRT‐IIIs are known for their central roles in diverse membrane remodeling activities in eukaryotic cells. This discovery, together with recent *in vitro* studies, now enables a molecular understanding of IM30/Vipp1 structure and activity in cyanobacteria and chloroplasts, an organelle of cyanobacterial origin. Here, we discuss membrane binding of IM30/Vipp1 in chloroplasts/cyanobacteria, contrasting them with eukaryotic ESCRT‐III function. Recent analyses have identified two key regions in IM30/Vipp1 that mediate initial membrane binding, as well as the formation of spiral, barrel, and/or rod structures on membrane surfaces, eventually facilitating membrane internalizations and tubulation. The potential roles of these membrane‐bound structures in the remodeling of chloroplasts and cyanobacterial membranes are discussed.

## Significance Statement

Although IM30/Vipp1 proteins were identified about two decades ago as essential for thylakoid membrane dynamics and their evolutionary relationship to bacterial PspA proteins was recognized early on, the recent discovery that these cyanobacterial and chloroplast proteins belong to the ESCRT‐III superfamily of membrane‐remodeling proteins has dramatically reshaped the field. This review highlights recent structural analyses of cyanobacterial and chloroplast Vipp1, consistently relating its structure and (proposed) functions to those of well‐characterized eukaryotic ESCRT‐III proteins.

## ESCRT PROTEINS ARE CONSERVED IN PRO‐ AND EUKARYOTES

Membrane remodeling and repair are essential for the biogenesis and maintenance of cellular and organellar membranes in pro‐ as well as in eukaryotes. A central player in diverse membrane remodeling and repair processes in the eukaryotic cytoplasm is the *endosomal sorting complex required for transport* (ESCRT) system (Henne et al., 2011; Hurley, 2015; Olmos, 2022; Vietri et al., 2020). Eukaryotic ESCRT systems comprise five major subunits: ESCRT‐0, ESCRT‐I, ESCRT‐II, ESCRT‐III, and the AAA‐ATPase Vps4. Yet, the critical membrane‐remodeling step is mediated by the ESCRT‐III subunits. This step involves the formation of large, hetero‐polymeric complexes. While all ESCRT subunits have multiple isoforms in eukaryotes, ESCRT‐III has the largest number of isoforms: eight in yeast, named *vacuolar protein sorting* (Vps), and 12 in human cells, called *charged multivesicular body protein* (CHMP) (Hurley, 2010; Pfitzner et al., 2021). The remaining eukaryotic ESCRT proteins assist in membrane recruitment of ESCRT‐IIIs. For example, ESCRT‐0 mediates binding of the ESCRT‐I and ESCRT‐II proteins, which act upstream of ESCRT‐III recruitment and are well characterized for their roles in initiating ESCRT‐III filament formation and generating membrane curvatures (Fyfe et al., 2011; Gill et al., 2007; Henne et al., 2011; Henne et al., 2012; Im et al., 2009). The downstream disassembly of eukaryotic ESCRT‐III filaments requires the AAA‐ATPase Vps4 (Babst et al., 1998), which appears to also facilitate membrane constriction during later stages of ESCRT‐mediated membrane remodeling (Maity et al., 2019; Pfitzner et al., 2020; Schöneberg et al., 2018).

ESCRT proteins are also encoded in the genomes of *Asgard* archaea, the closest evolutionary relatives of eukaryotes (Melnikov et al., 2025; Souza et al., 2025). Their genomes code for ESCRT‐II and ESCRT‐III proteins as well as for a Vps4‐like AAA‐ATPase (Hatano et al., 2022; Spang et al., 2015; Zaremba‐Niedzwiedzka et al., 2017). While the biological function of ESCRT‐III in *Asgard* archaea remains to be fully elucidated, the close evolutionary relationship between *Asgard* archaea and eukaryotes, as well as recent *in vitro* studies, suggests that isolated *Asgard* ESCRT‐III proteins are able to deform membranes (Melnikov et al., 2025; Souza et al., 2025). Therefore, the *Asgard* ESCRT‐III proteins are likely also involved in membrane remodeling processes *in vivo*. Beyond *Asgard* archaea, ESCRT‐III homologs are found throughout the TACK (*Thaumarchaeota*, *Aigarchaeota*, *Crenarchaeota*, and *Korarchaeota*) superphylum, where they are called Cdv (cell division) proteins (Caspi & Dekker, 2018; Ithurbide et al., 2022).

In 2021, structural analyses have revealed that ESCRT‐III proteins are not only present in eukaryotes and archaea but also in bacteria, demonstrating that the proteins and protein complexes they form, as well as their membrane remodeling activities, are evolutionarily conserved (Gupta et al., 2021; Junglas et al., 2021; Liu et al., 2021). Yet, in contrast to all other lineages, the ESCRT system appears to be far less complex in bacteria, as solely genes coding for ESCRT‐III proteins appear to be highly conserved (Carlton & Baum, 2023; Nachmias et al., 2025; Olmos, 2022; Williams & Low, 2025).

## ESCRT‐III PROTEINS IN (CYANO)BACTERIA AND CHLOROPLASTS

In bacteria, only a limited number of ESCRT‐III superfamily members are known: *phage shock protein A* (PspA), LiaH, YjfJ, and the *inner membrane‐associated protein of 30 kDa* (IM30), later also called the *vesicle‐inducing protein in plastids 1* (Vipp1). While PspA is encoded in multiple bacterial lineages, LiaH occurs primarily in Gram‐positive bacteria of the genus *Bacillus*, YjfJ in enterobacteria, and IM30/Vipp1 is present in cyanobacteria as well as in chloroplasts, an eukaryotic organelle with cyanobacterial origin (Jovanovic et al., 2014; Liu et al., 2021).

While still not completely understood, the bacterial ESCRT‐III protein PspA is currently best studied, mainly in *Escherichia coli*. PspA appears to be a key effector in a bacterial stress response system (Darwin, 2005; Joly et al., 2010) where it is involved in maintaining the transmembrane proton gradient (Joly et al., 2010; Kobayashi et al., 2007; Manganelli & Gennaro, 2017). In its soluble form, *E. coli* PspA is inactive, and the protein activity appears to be coupled to binding to the negatively charged surface of the bacterial inner membrane (Jovanovic et al., 2014). Additionally, PspA of the cyanobacterium *Synechocystis* sp. PCC 6803 (from here on: *Synechocystis*) has been shown to remodel membranes *in vitro*, which likely involves fusion/fission events, suggesting that PspAs perform a comparable function *in vivo* (Herianto et al., 2025; Hudina et al., 2025; Junglas et al., 2021; Junglas, Hudina, et al., 2025).

In *Bacillus subtilis*, LiaH is part of the LiaFSR system, which is involved in bacterial stress response (Mascher et al., 2004). Under non‐stressed conditions, LiaH is distributed in the cytoplasm. Upon activation of the LiaFSR system, LiaH is recruited to the cytoplasmic membrane with the help of the membrane‐anchoring protein LiaI, resulting in their colocalization. Membrane‐associated LiaH is assumed to stabilize the inner leaflet of the *Bacillus* cytoplasmic membrane, thereby helping to maintain the membrane integrity under stress (Domínguez‐Escobar et al., 2014).

Not much is known yet about enterobacterial YjfJ. Structural predictions indicate that it has an ESCRT‐III‐like homo‐polymeric structure, suggesting that it interacts with membranes as well (Rychel et al., 2025). PspA and YjfJ have both been shown to be involved in the stress response in *E. coli*, and YjfJ appears to stabilize and/or repair bacterial cytoplasmic membranes, similar to PspA (Rychel et al., 2025).

In 1994, a protein has been identified in pea chloroplasts that is associated with the inner envelope membrane as well as thylakoid membranes (TMs) (Li et al., 1994). Yet, the physiological function of this *inner membrane‐associated protein of 30 kDa* (IM30) remained elusive for a long time. Based on studies in *Arabidopsis thaliana* (from here on: *Arabidopsis*) and *Synechocystis* depletion strains, IM30 has later been shown to be essential in chloroplasts and cyanobacteria, where it was suggested to be somehow involved in the biogenesis and/or maintenance of TMs (Fuhrmann, Gathmann, et al., 2009; Kroll et al., 2001; Westphal et al., 2001). As initial observations in cold‐stressed *Arabidopsis* chloroplasts indicated an involvement in the formation of inner envelope membrane vesicles, the protein has been renamed to *vesicle inducing protein in plastids 1* (Vipp1) (Kroll et al., 2001). Yet, since the vesicle‐inducing function initially attributed to IM30 has not been consistently demonstrated since its first description, the term IM30 has continued to be used alongside Vipp1. More recently, however, IM30 has been adopted as an overarching term encompassing the entire group of bacterial ESCRT‐III‐related proteins, including PspA, Vipp1, and LiaH, reflecting a broader functional and evolutionary classification. This expanded usage now complicates equating IM30 solely with Vipp1. Notably, recent studies have shown that IM30 can induce vesicle formation *in vitro* under defined conditions (Junglas, Kartte, et al., 2025), as further discussed below. To avoid ambiguity and maintain conceptual clarity, we henceforth use the term Vipp1 to refer specifically to the protein originally described as IM30.

As an *in vivo* vesicle‐inducing activity was not further demonstrated in the years following its identification, Vipp1 has mainly been implicated in membrane repair events that occur under stress conditions, similar to PspA, visible, for example, by an increased heat stress tolerance observed upon overexpression of *vipp1* in *Arabidopsis* (Zhang et al., 2016). In fact, the Vipp1‐coding gene likely has evolved from *pspA* via gene duplication, a process that probably happened in a cyanobacterial progenitor, as cyanobacteria typically still contain both PspA and Vipp1, whereas photosynthetic organisms harboring chloroplasts exclusively code for Vipp1.

Vipp1 has been shown to form carpet structures on membranes that potentially stabilize membranes and block proton leakage (Junglas et al., 2020). This activity likely mirrors the membrane‐repair strategy of some eukaryotic ESCRT‐III proteins: Vipp1 is thought to be recruited to damaged membrane regions to stabilize and/or seal the membrane (Siebenaller et al., 2020). In line with this, Vipp1 redistributes from the cytoplasm to damaged membrane zones in cyanobacterial cells upon induced membrane damage (Gates et al., 2022). In addition, the protein preferentially localizes at highly curved TM regions that are prone to membrane defects (Gutu et al., 2018). Yet, the exact mechanisms by which PspA and Vipp1 stabilize and/or repair internal membranes in (cyano)bacteria and chloroplasts are currently only rudimentarily understood. Recent experimental analyses indicate that the proteins might seal membranes via patching or shedding processes, as further discussed below.

In recent years, many of the PspA/Vipp1 membrane remodeling activities have been elucidated based on studies with isolated cyanobacterial proteins. *In vitro*, cyanobacterial PspA and Vipp1 induce liposome fusion (Hennig et al., 2015; Junglas et al., 2021) and also tubulate membranes, eventually resulting in the formation of vesicular structures (Junglas et al., 2021; Hudina et al., 2025; Junglas & Hudina et al., 2025; Junglas, Kartte, et al., 2025). Thus, both eukaryotic ESCRT‐III complexes and their prokaryotic counterparts execute membrane remodeling as well as membrane repair functions, potentially via membrane excision or patching of damaged membrane sites. Importantly, the formation of TMs in chloroplasts has been shown to involve fusion of vesicular structures budding from the chloroplast inner envelope membrane (Wettstein, 1960), and dynamic membrane remodeling has been shown to involve fusion and fission of TM stacks in chloroplasts (Chuartzman et al., 2008). Based on the now available *in vitro* observations, all these processes can well be driven by Vipp1 and/or PspA in cyanobacteria and chloroplasts.

## THE STRUCTURE OF ESCRT‐III PROTEINS

**Figure 1:**
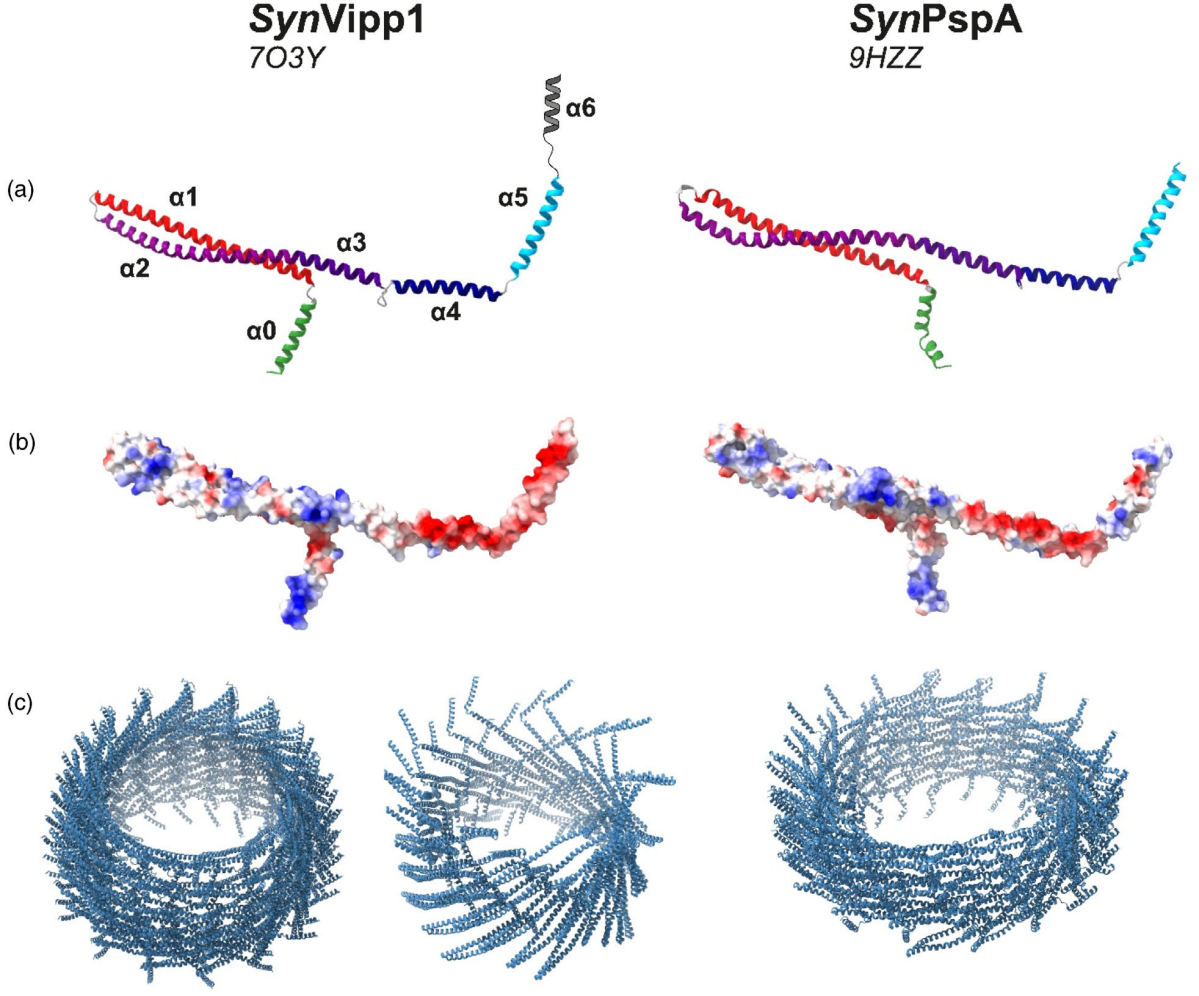
Structure and electrostatic surface distribution in Vipp1/PspA mono‐ and polymers. (a) Monomeric structures of Vipp1 and PspA proteins of *Synechocystis* sp. PCC 6803 (*Syn*Vipp1, *Syn*PspA) with differently colored helices and (b) electrostatic surface potential. (c) Polymeric structures of Vipp1 and PspA proteins. For Vipp1, a C16 symmetric barrel (left) and a tube‐like assembly (right) (8QHV). For PspA, four turns of a polymeric PspA helix are shown.

Despite low primary‐sequence identity, ESCRT‐III proteins from eukaryotes, archaea, and bacteria are all predominantly α‐helical and share a conserved secondary structure composed of five α‐helices connected by flexible hinge regions (Williams & Low, 2025). The structural core of this conserved ESCRT‐III fold is a helical hairpin formed by helices α1 and α2 (Schlösser et al., 2023). Furthermore, all bacterial and some eukaryotic ESCRT‐III proteins possess an additional N‐terminal helix α0 that has been associated with membrane binding (Buchkovich et al., 2013; Gupta et al., 2021; Huber et al., 2020; Jovanovic et al., 2014; Junglas et al., 2021; Liu et al., 2021; McDonald et al., 2015; McDonald et al., 2017; Otters et al., 2013; Schlösser et al., 2023), as further discussed below. Besides the five prototypical helices, eukaryotic ESCRT‐IIIs typically contain extra C‐terminal domains, many of which are involved in protein–protein interactions (Nguyen et al., 2020; Obita et al., 2007; Shim et al., 2007; Tang et al., 2015). In contrast to other bacterial ESCRT‐III proteins, Vipp1 of cyanobacteria and chloroplasts are C‐terminally prolonged by a disordered region followed by a (predicted) α‐helix (Figure 1a). While the exact function of this region is still enigmatic, it seems to be advantageous for the Vipp1 *in vivo* functions in chloroplasts and cyanobacteria (Hennig et al., 2017; Ma et al., 2025). Moreover, helix α6 has been proposed to contribute to Vipp1 membrane binding (Hennig et al., 2017) and potentially negatively regulates *At*Vipp1 supercomplex formation (Zhang et al., 2016) through interactions with the chloroplast Hsp70 system (Li et al., 2025).

All ESCRT‐III superfamily members analyzed thus far form diverse polymeric structures. In eukaryotes, different ESCRT‐III subunits typically hetero‐polymerize, although several members, such as Vps24, Shrub, CHMP1B, and IST1, are also capable of forming homo‐polymers, at least *in vitro* (Huber et al., 2020; McCullough et al., 2015; McMillan et al., 2016; Nguyen et al., 2020). *Asgard* ESCRT‐IIIs also form both homo‐ and hetero‐polymeric structures *in vitro* (Melnikov et al., 2025; Souza et al., 2025).

In contrast, solely homo‐polymeric structures have been reported so far for the crenarchaeal ESCRT‐III protein CdvB2 and for bacterial ESCRT‐III proteins (Drobnič et al., 2025; Gupta et al., 2021; Junglas et al., 2021; Liu et al., 2021). Nevertheless, crenarchaeal ESCRT‐IIIs potentially also hetero‐polymerize, as CdvB2 could hetero‐polymerize with CdvB, and cyanobacteria contain two ESCRT‐III superfamily members, PspA and Vipp1, raising the possibility of mixed‐subunit polymers (Drobnič et al., 2025; Liu et al., 2021). The two Vipps identified in chloroplasts of the green algae *Chlamydomonas reinhardtii* (from here on: *Chlamydomonas*), Vipp1 and Vipp2, have been shown to interact, indicating the formation of hetero‐polymers (Theis et al., 2020). Yet, the formation of hetero‐polymeric assemblies still needs to be proven.

Eukaryotic and archaeal ESCRT‐III proteins, *E. coli* PspA and *Chlamydomonas* Vipp1, as well as *Nostoc punctiforme* and *Synechocystis* PspA assemble into tubular rod structures (Bertin et al., 2020; Drobnič et al., 2025; Effantin et al., 2013; Huber et al., 2020; Junglas et al., 2021; Liu et al., 2007; Liu et al., 2021; Male et al., 2014; McCullough et al., 2015; McMillan et al., 2016; Melnikov et al., 2025; Moriscot et al., 2011; Nguyen et al., 2020; Souza et al., 2025; Theis et al., 2019). Moreover, Vipp1 of cyanobacteria, *Chlamydomonas, Arabidopsis*, and *Triticum urartu*, as well as *E. coli* PspA and LiaH of *Bacillus subtilis* (additionally), form barrel structures (Aseeva et al., 2004; Fuhrmann, Bultema, et al., 2009; Gao et al., 2015; Gao et al., 2017; Gupta et al., 2021; Hankamer et al., 2004; Liu et al., 2021; Saur et al., 2017; Wolf et al., 2010). Cyanobacterial Vipp1 is dispersed throughout the cytoplasm and forms localized assembly states, which are more pronounced when cells are stressed (Bryan et al., 2014; Gates et al., 2022; Quarta et al., 2026). Similarly, Vipp1 in chloroplasts is localized in distinct *puncta* structures (Nordhues et al., 2012; Zhang et al., 2012; Zhang et al., 2016). While the exact nature of the formed structures is currently unclear, the proteins appear to assemble close to internal membranes, especially TMs, in line with a proposed membrane stabilizing and/or repair function. Furthermore, when highly overexpressed, Vipp1 of *Arabidopsis* has been shown to form extended rod structures in unstressed tobacco plant chloroplasts (Gachie et al., 2025), similar to the observations made with Vipp1 in *Chlamydomonas* (Gupta et al., 2021).

**Figure 2:**
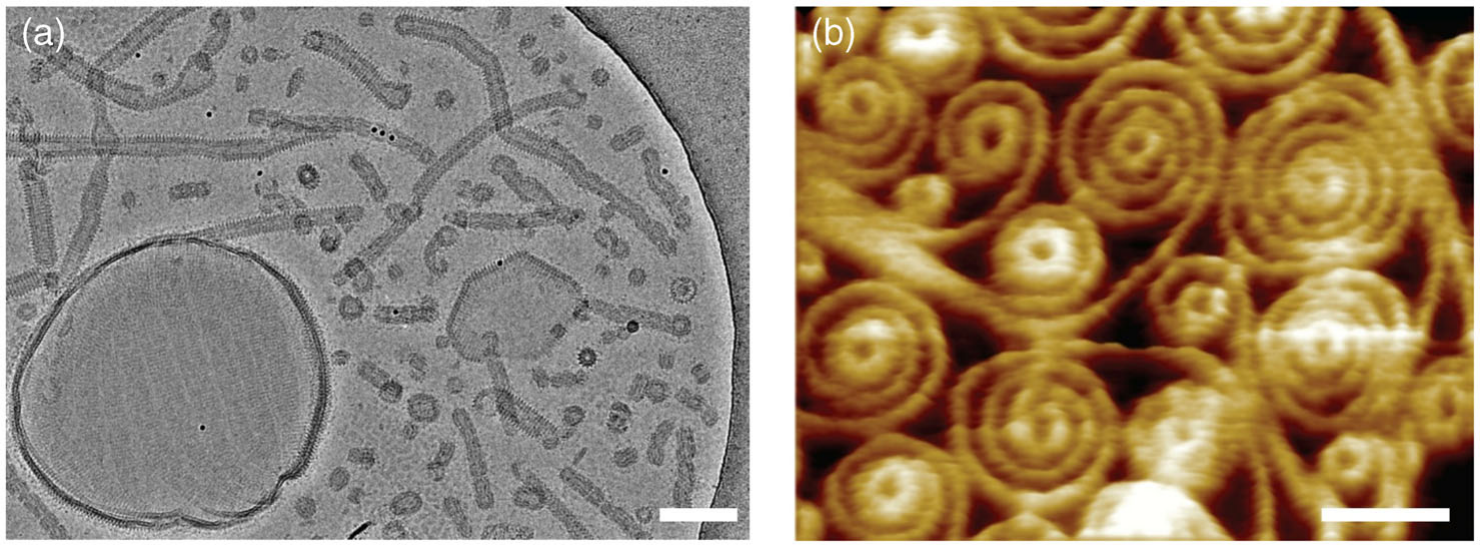
Diverse membrane‐bound structures of cyanobacterial Vipp1. (a) Cryo‐EM micrograph showing a variety of *Synechocystis* Vipp1 assemblies formed after reconstitution in the presence of lipids. Vipp1 organizes into flat, membrane‐associated structures, such as carpets and loose coats, as well as into ring‐shaped, stacked‐ring, and various tubular assemblies that have internalized membrane tubes (Junglas, Kartte, et al., 2025). Scale bar = 100 nm. (b) Representative F‐AFM height image of Vipp1 from *Nostoc punctiforme* bound to a supported lipid bilayer, revealing diverse flat polymeric structures, including sheets, spirals, and rings (Naskar et al., 2025). Scale bar = 100 nm. The research original articles presenting the figures are licensed under a Creative Commons Attribution 4.0 International License. To view a copy of this license, visit http://creativecommons.org/licenses/by/4.0/.

Vipp1 barrel/rod structures have been shown to bind to solid‐supported membrane surfaces, accompanied by polymer disassembly (Junglas et al., 2020). Yet, membrane binding does not require preformed large polymeric assemblies, as Vipp1 monomers or smaller oligomers also bind with high affinity to membrane surfaces (Heidrich et al., 2016; Junglas et al., 2020; Schlösser et al., 2025). Upon membrane binding, Vipp1 proteins can form sheets or membrane surface‐covering carpets (Figure 2a,b) (Junglas, Kartte, et al., 2025; Naskar et al., 2025; Pan et al., 2025). Furthermore, when assembling on flat membranes, eukaryotic ESCRT‐IIIs, as well as cyanobacterial Vipp1s, have been shown to form spiral structures (Bertin et al., 2020; Jukic et al., 2022; Jukic et al., 2023; Liu et al., 2024; McCullough et al., 2015; Naskar et al., 2025; Pan et al., 2025), likely an early step in membrane deformation and potentially fission (Chiaruttini et al., 2015). Cyanobacterial PspA and Vipp1 homo‐polymeric assemblies (Figure 1c) can also tubulate membranes via the formation of large, homo‐polymeric barrel and/or rod structures on a membrane surface coupled with membrane internalization (Figure 2a) (Hudina et al., 2025; Junglas et al., 2021; Junglas, Hudina, et al., 2025; Junglas, Kartte, et al., 2025).

While all thus far analyzed ESCRT‐III superfamily members assemble into large polymeric structures/filaments and can remodel membranes, at least *in vitro*, the exact mechanism of how the ESCRT systems mediate membrane remodeling *in vivo* is still under debate.

## MEMBRANE RECRUITMENT OF ESCRT‐III PROTEINS

In eukaryotes, monomeric ESCRT‐III proteins are localized in the cytoplasm and recruited by targeting factors that direct eukaryotic ESCRT components to a variety of different intracellular membranes, including the plasma membrane, nuclear envelope, endolysosomal compartments, and autophagosomes (Olmos, 2022; Vietri et al., 2020). Cellular functions mediated by eukaryotic ESCRT‐III proteins at these membranes fall broadly into two classes: membrane remodeling/fission and membrane repair, yet these functions might be linked.

During membrane remodeling events, such as viral budding, microvesicle formation, and cytokinetic abscission, ESCRT‐III is recruited to membranes through distinct adaptor proteins that mediate site‐specific assembly (Baumgärtel et al., 2011; Carlton & Martin‐Serrano, 2007; Lee et al., 2008; Martin‐Serrano et al., 2001; Morita et al., 2007; Nabhan et al., 2012; Votteler & Sundquist, 2013). Several membrane repair mechanisms depend on the recruitment of ESCRT‐III proteins to rapidly seal damaged cellular membranes. Proper ESCRT‐III recruitment is, for example, critical during nuclear envelope repair and at mitotic exit, where ESCRT‐IIIs help reseal the membrane to ensure cellular integrity and proper cell division (Halfmann et al., 2019; Jimenez et al., 2014; Olmos et al., 2016; Radulovic et al., 2018; Scheffer et al., 2014; Skowyra et al., 2018; Sønder et al., 2019; Vietri et al., 2015; von Appen et al., 2020).

Thus, multiple mechanisms exist in the cytoplasm of eukaryotic cells by which ESCRT‐III proteins are recruited to intracellular membranes. However, eukaryotic ESCRT‐IIIs can deform membranes in the absence of other ESCRT proteins *in vitro*, indicating that the isolated ESCRT‐III subunits (i) have the propensity to directly bind to membrane surfaces and (ii) are capable of remodeling membranes in the absence of any other ESCRT subunit.

Similar to eukaryotic membrane‐targeting factors, in the currently best‐studied *E. coli* PspA system, PspBC is involved in recruiting PspA to membranes (Adams et al., 2003; Darwin, 2005). However, isolated *Eco*PspA also binds well to bacterial cytoplasmic membranes even in the absence of PspBC (Kobayashi et al., 2007; McDonald et al., 2015). Accordingly, the cyanobacterial and chloroplast ESCRT‐IIIs, PspA and Vipp1, bind well to membranes without the need of any intermediary factors (Heidrich et al., 2016; Hennig et al., 2015; Herianto et al., 2025; Hudina et al., 2025; Junglas et al., 2021; Junglas, Hudina, et al., 2025; Junglas, Kartte, et al., 2025; Schlösser et al., 2025). Yet, a number of Vipp1‐interacting proteins have been identified recently that could serve as membrane‐targeting factors (Kreis et al., 2023; Yilmazer et al., 2025), as further discussed below.

In some eukaryotic ESCRT‐III proteins, including yeast Snf7, as well as the prokaryotic homologs Vipp1 and PspA, the amphipathic helix α0 appears to function as a membrane anchor (Buchkovich et al., 2013; Hudina et al., 2025; Jovanovic et al., 2014; Junglas, Kartte, et al., 2025; McDonald et al., 2015; McDonald et al., 2017; Otters et al., 2013). Crenarchaeal ESCRT‐III paralogs likewise harbor an N‐terminal amphipathic helix that has been shown to be crucial for membrane association (Drobnič et al., 2025). Noteworthy, when present, the N‐terminal helix α0 typically is much shorter in eukaryotic ESCRT‐IIIs. However, some eukaryotic and archaeal ESCRT‐III proteins lack a canonical amphipathic α‐helix yet contain an N‐terminal segment enriched in hydrophobic and positively charged residues, which is predicted to contribute to membrane binding (Azad et al., 2023; Buchkovich et al., 2013; Melnikov et al., 2025). Pro‐ and eukaryotic ESCRT‐III supramolecular assemblies bind tubulated membranes, either on the inner or the outer surface of the ESCRT‐III filaments. The membrane‐interacting interface is located on the outside of the helical assembly for CHMP2A/CHMP3 (Azad et al., 2023), but on the inside for all other tubular ESCRT‐III proteins, the structures of which have been solved with bound membranes (Junglas, Hudina, et al., 2025; Junglas, Kartte, et al., 2025; Melnikov et al., 2025; Naskar et al., 2025; Nguyen et al., 2020; Souza et al., 2025). Yet, in all cases, the N‐terminal regions are crucial for membrane binding. However, eukaryotic ESCRT‐IIIs that do not contain an extended N‐terminal segment also assemble on membrane surfaces (Nguyen et al., 2020). This might be due to (i) co‐assembly with other eukaryotic ESCRT‐IIIs that contain an N‐terminal membrane‐binding segment on membrane surfaces, (ii) membrane recruitment via accessory (ESCRT) proteins, or (iii) membrane binding via other protein regions. In fact, studies with isolated pro‐ and eukaryotic ESCRT‐III proteins have indicated an involvement of the hairpin structure formed by helices α1/2 in membrane adhesion (Buchkovich et al., 2013; Herianto et al., 2025; Muzioł et al., 2006; Schlösser et al., 2025). Thus, multiple membrane‐binding interfaces are involved in membrane interaction, most notably the N‐terminal amphipathic helix α0 and the α1/2 region.

## STRUCTURE AND FUNCTION OF THE VIPP1 AND PspA HELIX α0

**Figure 3:**
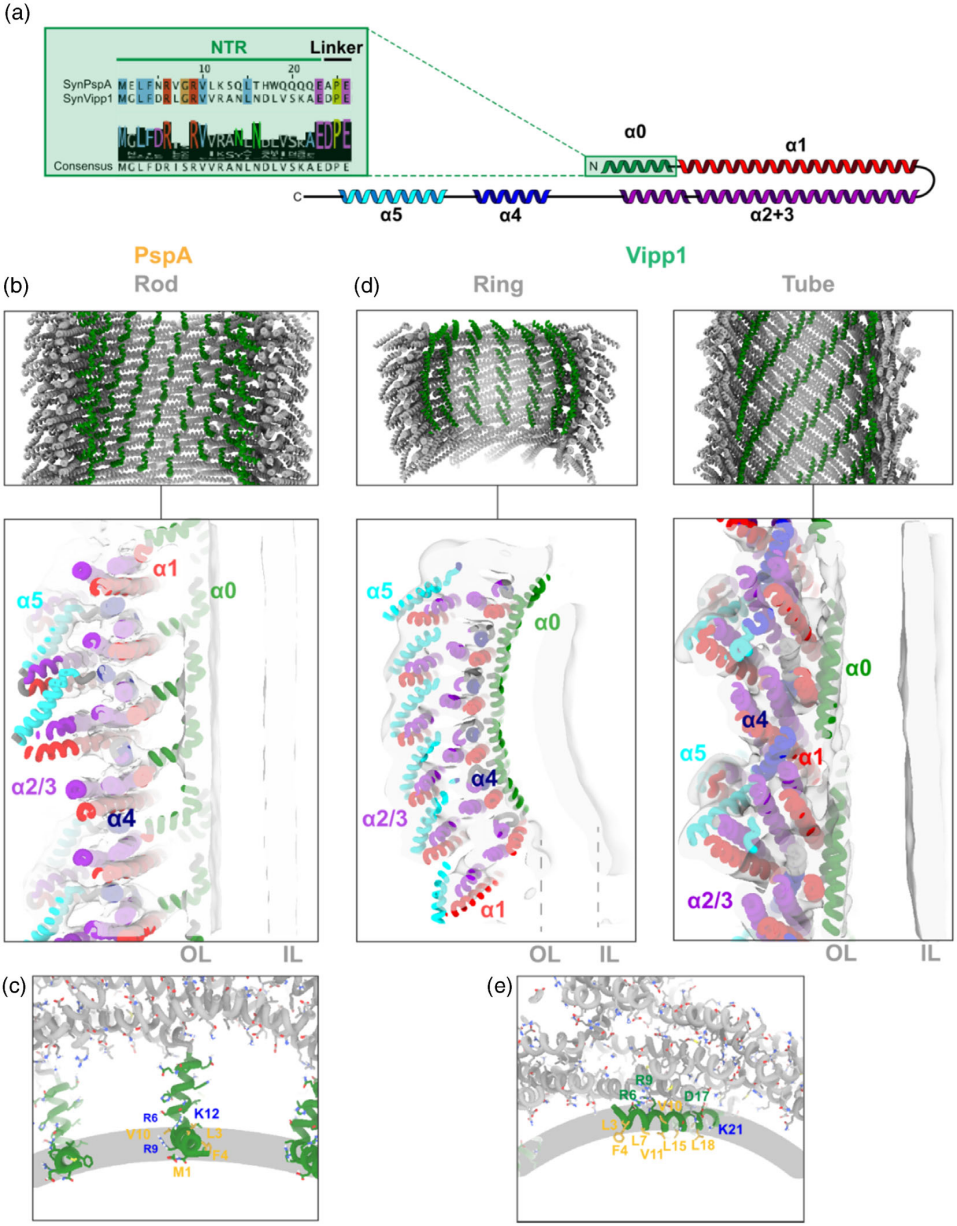
Helix α0 in *Syn*PspA and *Syn*Vipp1 assemblies. (a) Overview of the bacterial ESCRT‐III topology and sequence alignment of α0 from 250 sequences of a *Syn*PspA search query. The resulting consensus sequence together with the *Syn*PspA and *Syn*Vipp1 was visualized in Jalview (Waterhouse et al., 2009). Residues with >70% identity are colored by their properties according to the ClustalX color scheme. (b) Top: Overview of the *Syn*PspA rod structure with α0 highlighted in green (PDB: 9HZZ). Bottom: Enlarged view of the *Syn*PspA membrane interaction (PDB: 9HZZ, EMD‐15507). OL: outer leaflet, IL: inner leaflet. (c) Enlarged view of the molecular details and interactions of *Syn*PspA α0 (PDB: 9HZZ). Residues potentially interacting with the lipid acyl chains are colored in yellow. Residues potentially interacting with the lipid headgroups are colored in blue. (d) Top: Overview of *Syn*Vipp1 assembly structures with helix α0 highlighted in green (ring PDB: 7O3W, tube PDB: 8QHV). Bottom: Enlarged view of Vipp1 membrane interaction (ring PDB: 7O3W EMD‐18425, tube PDB: 8QHV EMD‐18420). (e) Enlarged view of the molecular details and interactions of the Vipp1 helix α0 (PDB: 9EOP). Residues potentially interacting with the lipid acyl chains are colored in yellow. Residues potentially interacting with the lipid headgroups are colored blue. Residues interacting with the protein scaffold are colored green.

Bacterial PspA and Vipp1 proteins contain an N‐terminal extension of 20–30 amino acids, which has the potential to form an amphipathic α‐helix. In the absence of lipids, this region is unstructured; however, it will be referred to as helix α0 for clarity and to avoid inconsistencies in terminology (Hudina et al., 2025; Junglas et al., 2021; Junglas, Kartte, et al., 2025; McDonald et al., 2017; Osadnik et al., 2015). The helix starts with an evolutionarily conserved MGLFDRxxRV motif, followed by a more variable segment enriched in hydrophobic residues (Junglas et al., 2021). Helix α0 is connected to the remainder of the protein through a linker region containing the conserved EDPE motif (Figure 3a). Only upon lipid binding, the α0 sequence adopts an amphipathic α‐helical conformation (α0) (Hudina et al., 2025; Junglas, Hudina, et al., 2025; McDonald et al., 2017). Accordingly, the isolated helices α0 of *E. coli* and *Synechocystis* PspA as well as of *Synechocystis* Vipp1 interacts with negatively charged membranes, sense and induce membrane curvature, and likely function as a membrane anchor (Gupta et al., 2021; Hudina et al., 2025; Jovanovic et al., 2014; Junglas, Hudina, et al., 2025; Junglas, Kartte, et al., 2025; McDonald et al., 2015; McDonald et al., 2017). For both cyanobacterial PspA and Vipp1, membrane interaction of the amphipathic helix α0 critically depends on defined hydrophobic and positively charged residues (Gupta et al., 2021; Hudina et al., 2025; Junglas, Hudina, et al., 2025; Junglas, Kartte, et al., 2025; McDonald et al., 2017).

Despite the shared sequence features of the cyanobacterial ESCRT‐III homologs PspA and Vipp1, the determined structures with tubulated membranes revealed that the conformation and membrane interaction mode of helix α0 differ in detail (Hudina et al., 2025; Junglas, Kartte, et al., 2025; Naskar et al., 2025). In *Syn*PspA rods, α0 helices line up and face the lumen of the rods, resulting in an even spacing (Figure 3b). Here, helix α0 is kinked at Gly8, with the part N‐terminal to the kink being in contact with the membrane and the part C‐terminal to the kink serving as a spacer between the membrane and the protein rod. As a result, helix α0 maintains a constant ~35 Å gap between the protein rod and the membrane tube across all diameters of PspA rods (Hudina et al., 2025). This gap enables exclusively helix α0 to directly interact with the membrane within PspA rods, while it separates the rest of the protein from the lipid surface. In the rod lumen, each helix α0 appears to be completely independent, without any direct contact with neighboring α0 helices or other PspA parts. However, as the entire helix α0 can bind to membrane surfaces (Hudina et al., 2025), it has been hypothesized that during membrane tubulation by PspA, helix α0 initially is completely embedded in the outer leaflet of the membrane. In the later tubulation phase, this helix is partially pulled away from the membrane during formation of the polymer scaffold, leaving only the N‐terminal part with the strongest interaction in contact with the membrane (Hudina et al., 2025). The N‐terminal part is submerged in the headgroup area of the outer leaflet (OL) of the engulfed membrane tube. Here, Arg6, Arg9, and Lys12 have been shown to interact with negatively charged lipid head group regions in the OL, which is critical for membrane binding of helix α0 (Hudina et al., 2025), while the hydrophobic face of the PspA helix α0 (Met1, Leu3, Phe4, Val7, Val10) likely interacts with the hydrophobic membrane core (Figure 3c). During membrane tubulation, the energy released by the additive interactions of multiple α0 helices with the membrane likely compensates for the energy required to induce membrane curvature. The progressive polymerization of PspA subunits on the membrane surface leads to an increased curvature of the membrane; as more α0 helices interact with the membrane, more energy is gained, and a pulling force is generated that finally leads to the formation of an emerging membrane tubule on the inside of the PspA assembly (Hudina et al., 2025).

In analogy to *Syn*PspAs lipid tubule structures, helix α0 faces the lipid tubule also in the lumen of *Syn*Vipp1 assemblies (Junglas, Kartte, et al., 2025). However, in Vipp1 polymers, helix α0 forms contiguous columns in the lumen (Figure 3d). Although the orientation and tilt of these columns change depending on the exact type of polymeric assembly, the spacing between the columns and the spacing between the individual α0 helices remains constant (Figure 3d). Furthermore, in the presence of membranes, helix α0 folds into a straight helix that is fully embedded into the headgroup region of the OL in all assembly types (Junglas, Kartte, et al., 2025), avoiding the gap between the membrane and the protein assembly, as observed for PspA. Consequently, the available membrane interaction surface is much larger in Vipp1 polymers compared to PspA, as the full‐length helix α0 as well as parts of the α1 and α2/3 (i.e., Lys27, Arg49, Lys144, Lys147) are exposed to the membrane headgroup region. Accordingly, the hydrophobic face of the Vipp1 helix α0 capable of interaction with the hydrophobic membrane core region in the tubulated membrane consists of 10 (Met1, Leu3, Phe4, Leu7, Val10, Val11, Leu15, Leu18, Val19, Ala22) instead of only five hydrophobic residues in PspA (Figure 3c,e). Replacement of the conserved hydrophobic residues Phe4 and Val11 severely impaired stress resistance and TM remodeling in living cyanobacteria (Gupta et al., 2021), underscoring the importance of the hydrophobic helix α0 face for the Vipp1 function.

Although the Vipp1 helix α0 contains four positively charged residues that could interact with negatively charged lipid headgroups, not all of these are available for membrane interaction. As seen in recent structures, in Vipp1 polymers, helix α0 is connected to the protein scaffold via interactions of Asp5, Arg6, Arg9, Arg12, Asp17, and Glu23 with residues located on helices α1 and α4, respectively (Gupta et al., 2021; Junglas, Kartte, et al., 2025; Naskar et al., 2025). Additionally, Arg6 and Arg9 interact with Glu23 and Asn16 from neighboring α0 helices. Thus, Arg6 and Arg9 are buried, and only Arg12 and Lys21 are exposed to the lipid headgroups (Figure 3e). Mutations of conserved helix α0 residues involved in inter‐monomer contacts bias Vipp1 toward rod formation (Gupta et al., 2021), and complete removal of the helix yields exclusively rod assemblies with PspA‐like symmetry for *Syn*Vipp1 (Junglas, Kartte, et al., 2025; Thurotte & Schneider, 2019). In contrast, truncation of helix α0 in *Chlamydomonas* Vipp1 led to the formation of fewer and smaller polymeric ring structures (Gao et al., 2015), while the same truncation completely prevented the assembly of large polymeric complexes of *Arabidopsis* Vipp1 (Otters et al., 2013). These data suggest that, in Vipp1 assemblies, helix α0 columns stabilize the assembly geometry through intra‐ and intermolecular interactions with α1 and α4, whereas in helix α0‐deficient assemblies, the altered monomer register and spacing prevent such stabilizing contacts.

Despite some differences, neither PspA nor Vipp1 assemblies can tubulate membranes within the assembly lumen without helix α0 (Hudina et al., 2025; Junglas, Kartte, et al., 2025), although an α0‐deficient *Syn*Vipp1 protein is still capable of binding to and remodeling membranes (Schlösser et al., 2025; Thurotte & Schneider, 2019). In contrast, *Arabidopsis* Vipp1 without helix α0 appears not to be able to associate with lipids or membranes (Otters et al., 2013). These observations, together with the structural data discussed above, suggest that helix α0 is an important membrane interactor and anchor of bacterial ESCRT‐III proteins within PspA and Vipp1 polymers and has an important role in stabilizing large PspA/Vipp1 polymeric structures. However, the observed membrane binding of α0‐deficient Vipp1 and PspA proteins (especially variants deficient in polymer formation) poses the question of whether other protein regions might (also) be relevant for membrane binding (Herianto et al., 2025; Thurotte & Schneider, 2019). Thus, the membrane interaction mode may differ between monomeric Vipp1/PspA and the diverse polymeric forms (spirals, barrels, rods). Regions of the proteins inaccessible in the polymers might interact with the membrane when the proteins are monomeric or form small oligomers. Particularly in the early stage of membrane tubulation, when the polymer is not yet fully formed, PspA/Vipp1 likely depends on a large membrane interaction surface (see below).

## STRUCTURE AND FUNCTION OF THE VIPP1 AND PspA HELIX α1‐3 HAIRPIN

All members of the ESCRT‐III superfamily share the presence of conserved basic residues within the α1–3 region. Yet, the exact location of these basic residues varies between individual proteins and organisms. In the yeast Snf7, several Lys residues (Lys60, Lys64, Lys68, Lys71, Lys79) on helix α2 and Lys112 and Lys115 on helix α3 were suggested to be relevant for membrane interaction (Buchkovich et al., 2013). In *Asgard* ESCRT‐IIIB, membrane binding is mediated primarily by helix α1, the N‐terminal region corresponding to α0, and the loop connecting helices α3 and α4, which contains an exposed patch of positively charged and hydrophobic residues (Souza et al., 2025). Hetero‐polymeric structures formed by the human CHMP2A and CHMP3 appear to employ basic residues located on helix α1 (Arg8, Lys9 (CHMP2A)) and within helices α3 and α4 (Lys104‐Lys136 (CHMP2A, CHMP3)) for membrane attachment (Azad et al., 2023). For CHMP3, however, conserved basic residues that could mediate membrane binding are presumed to reside in helix α1. Supporting this assumption, mutations of specific Lys and Arg residues (Arg24, Lys25, Arg28, Arg32, and Arg35) within helix α1 of CHMP3 abolish membrane binding (Muzioł et al., 2006). Similar positioning of conserved basic residues is inferred for CHMP1B/IST1 complexes as well as in the yeast CHMP3 analog Vps24 and in Vps2 (Buchkovich et al., 2013; Muzioł et al., 2006; Nguyen et al., 2020). These mechanistic insights from eukaryotic ESCRT‐III proteins align well with recent findings on cyanobacterial and chloroplast ESCRT‐IIIs. In *Syn*PspA, the basic residue Arg44 conserved in helix α1 appears to be of special importance for membrane interaction (Herianto et al., 2025). Given that Vipp1 is closely related to PspA and contains equivalent basic residues in corresponding positions, it is likely that this residue is also important for Vipp1 membrane binding in cyanobacteria and chloroplasts (Herianto et al., 2025). This notion is supported by recent molecular dynamics simulations of the isolated Vipp1 helix α1‐3 hairpin, which suggest that membrane interaction of this helical hairpin is primarily mediated via helix α1 upon contact with negatively charged membranes (Schlösser et al., 2025). However, in the polymeric PspA and Vipp1 assemblies, Arg44 engages in a salt bridge with Glu126, orienting it toward the protein core rather than the lumen of the polymer. As a result, the residue appears to be positioned too far from the membrane to participate in direct interaction. This potentially indicates structural rearrangements of the helix α1–3 hairpin structures associated with polymer formation.

The presence of basic residues in the α1–3 hairpin (Figure 1b) is in line with the general preference of ESCRT‐III proteins to bind to negatively charged membrane surfaces through electrostatic interactions (Buchkovich et al., 2013; Heidrich et al., 2016; Huber et al., 2020; Melnikov et al., 2025; Schlösser et al., 2025; Souza et al., 2025; Theis et al., 2019). Such interactions with negatively charged lipid membranes are thought to be essential for the nucleation and subsequent polymerization of both *Asgard* ESCRT‐IIIB and the eukaryotic ESCRT‐III protein Snf7 (Chiaruttini et al., 2015; Souza et al., 2025). How polymerization and polymer disassembly are regulated off versus on membranes still is unclear, albeit chaperones appear to be involved in these processes (Bryan et al., 2014; Gao et al., 2015; Kreis et al., 2023; Li et al., 2025; Liu et al., 2007; Yilmazer et al., 2025).

Studies using various truncated variants have demonstrated that helix α1 of *Syn*PspA and the α1‐3 hairpin of *Syn*Vipp1 alone are sufficient for membrane binding (Herianto et al., 2025; Schlösser et al., 2025; Thurotte & Schneider, 2019). In solution, the Vipp1 α1‐3 hairpin is folded, whereas the α0 and α4‐6 regions are unfolded in the monomeric or low molecular‐weight form, and either polymerization or membrane association is required for these helices to fold properly (Junglas et al., 2020; McDonald et al., 2017; Quarta et al., 2024; Thurotte & Schneider, 2019). Upon membrane binding, the structure of the α1‐3 hairpin rearranges: bending of the hairpin exposes part of the helical structure and partially unfolds the C‐terminal segments of helix α3 (Schlösser et al., 2025). The extent of these structural rearrangements appears to depend on the protein's surface density in a biphasic manner: the structural changes get more pronounced until all protein is membrane bound. The structural alterations decrease with increasing available membrane surface area, likely due to decreased protein–protein contacts (Schlösser et al., 2025). The influence of the surface protein density on membrane binding has also been observed for Snf7: on membranes densely coated with Snf7, polymers cease to grow and form polygonal structures, whereas at lower surface densities, the protein preferentially assembles into spirals (Chiaruttini et al., 2015).

In addition to the conserved electrostatic interactions between the basic amino acids and the negatively charged membrane surface, hydrophobic residues within the hairpin likely contribute to membrane stabilization. In the unbound state, these residues are involved in maintaining and stabilizing the helical hairpin structure through leucine zipper‐type interactions. Upon membrane association, some of these residues might interact with the hydrophobic core region of the membrane bilayer following initial electrostatic interactions mediated by the basic residues (Schlösser et al., 2025). The structural rearrangements observed within the α1–3 region could also underlie larger structural rearrangements in the full‐length protein upon membrane binding (Schlösser et al., 2025).

Although helices α1–3 appear to be crucial for membrane binding of ESCRT‐III proteins across all domains of life, helix α0 clearly enhances membrane binding (Buchkovich et al., 2013; Herianto et al., 2025; Schlösser et al., 2025). In the case of eukaryotic Snf7, the role of helix α0 is controversially discussed. Some studies suggest that the N‐terminal membrane‐anchoring helix α0 further stabilizes Snf7 on membranes, while membrane association is primarily mediated by α1–3 (Buchkovich et al., 2013; Tang et al., 2015). Another report, however, proposes that Snf7 employs helix α0 as a stable membrane anchor, with the α2/3 region oriented outward, unable to contact the membrane directly (Liu et al., 2024). These discrepancies may arise from differences in the polymeric state and/or the experimental setup, resulting in different membrane‐bound states observed. The latter protein conformation was observed on flat membranes (Liu et al., 2024), whereas the former was detected on curved membranes (Buchkovich et al., 2013; Tang et al., 2015). This assumption aligns with other observations, such as *Asgard* ESCRT‐IIIA binding exclusively to curved, but not flat, membranes (Souza et al., 2025) and with the finding that Vipp1 forms distinct assemblies on supported lipid bilayers depending on whether it interacts with membrane patches or fully covered surfaces (Naskar et al., 2025; Pan et al., 2025), which appear to differ from the polymeric assemblies it forms on liposomes (Junglas, Kartte, et al., 2025) (Figure 2).

Together, these observations suggest that multiple membrane‐bound states can exist, mediated by helix α0, the α1–3 helical hairpin, or a combination of both. The dominant binding mode likely depends on different factors involving the protein's polymeric state, membrane curvature (flat *vs*. curved), the degree of membrane perturbation, and the content of negatively charged lipids. Furthermore, the ability to switch between the α0‐ and α1–3‐mediated membrane interfaces may play an important role during remodeling of cyanobacterial and chloroplast inner membranes.

## DIFFERENT PROTEIN REGIONS MEDIATE DISTINCT STEPS DURING CYANOBACTERIAL/CHLOROPLAST ESCRT‐III MEMBRANE REMODELING

In PspA and Vipp1, two distinct membrane‐interacting regions have now been identified. The isolated α0 helices of cyanobacterial PspA and Vipp1 have been experimentally shown to interact with membrane surfaces (Hudina et al., 2025; Junglas, Kartte, et al., 2025; McDonald et al., 2017), and this interaction is critical for the formation and stabilization of membrane tubules within PspA and Vipp1 polymeric structures, such as rods or barrels (Hudina et al., 2025; Junglas, Kartte, et al., 2025). However, even in the absence of helix α0, PspA and Vipp1 stably associate with membranes yet are not capable anymore of tubulating membranes (Herianto et al., 2025; Hudina et al., 2025; Junglas, Kartte, et al., 2025). Furthermore, PspA and Vipp1 preformed polymeric structures appear not to be highly competent to internalize and tubulate membranes, but membrane tubulation appears to require the formation of polymeric assemblies on membrane surfaces from monomeric and/or lower oligomeric states (Junglas et al., 2021; Junglas, Kartte, et al., 2025). Assuming that monomers or smaller oligomers initially bind to the membrane and that helix α0 is only essential for membrane internalization in polymeric structures, but not for membrane interaction *per se*, suggests that initial membrane recruitment is not mediated by helix α0 (alone). Instead, the primary driver for the initial attachment of PspA/Vipp1 monomers to membranes potentially is the positively charged surface of the α1–3 helical hairpin (Figure 1b), as observed with the *Synechocystis* PspA and Vipp1 proteins (Herianto et al., 2025; Schlösser et al., 2025). For Vipp1 from *Arabidopsis*, the absence of helix α0 prevented binding to inner envelope vesicles prepared from pea chloroplasts (Otters et al., 2013). Similarly, PspA from *E. coli* did not bind well to the *E. coli* inner membrane when helix α0 was removed (Jovanovic et al., 2014), indicating that the α1–3 helical hairpin is not available or sufficient for membrane interaction in these cases.

**Figure 4:**
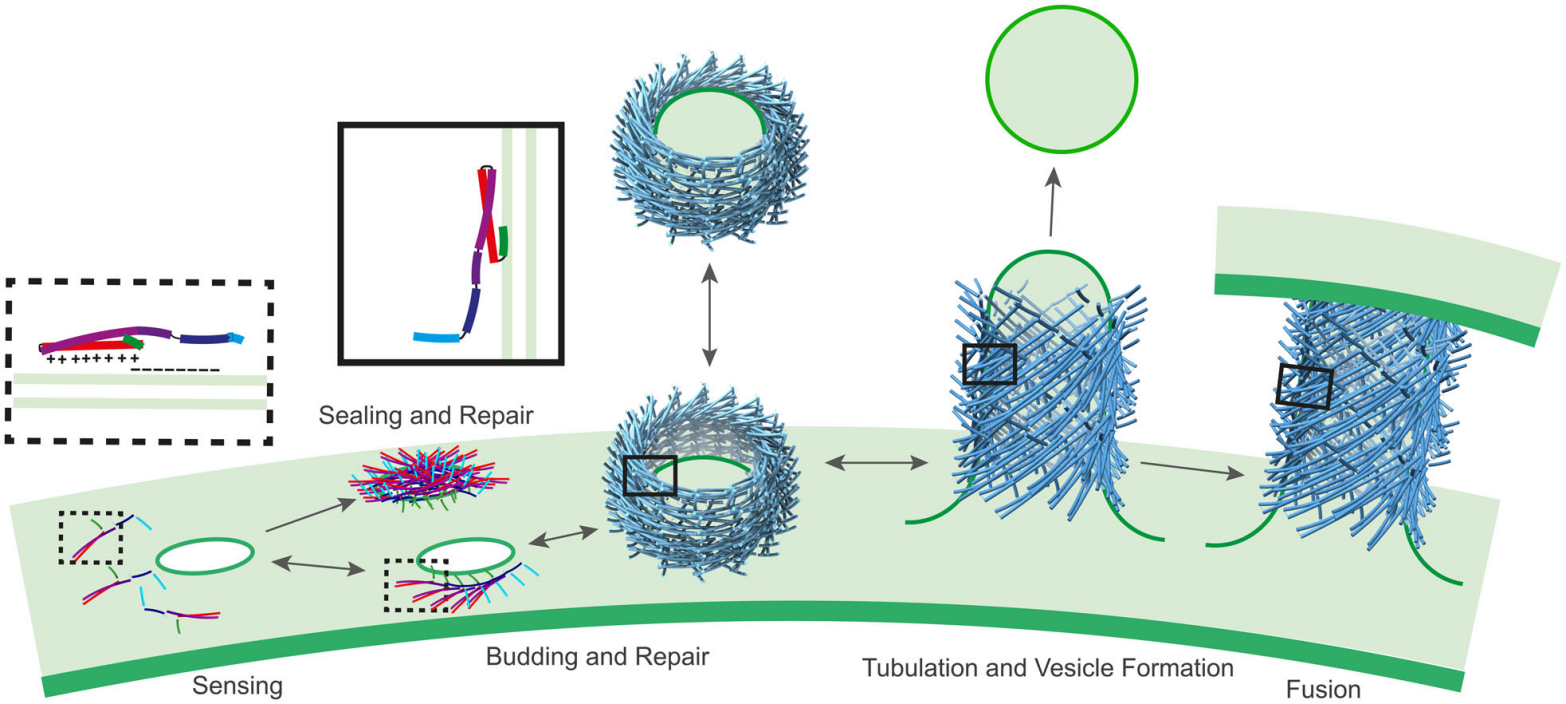
Vipp1 membrane binding and putative functions of membrane‐bound states. Model of membrane Vipp1 binding mediated by different protein regions and functions of membrane‐bound states: Initial membrane contact is primarily mediated by basic residues within the helical hairpin region (α1–3) via electrostatic interactions when Vipp1 is in a monomeric or low‐oligomeric state. Upon membrane binding, Vipp1 eventually polymerizes on a membrane surface, with helices α1–3 contributing to inter‐monomer contacts. The resulting flat assemblies participate in membrane sealing, patching, and repair and may serve as precursors for ring formation, which is essential for repair processes involving membrane shedding. Ring and spiral structures can transition into membrane‐internalizing tubular assemblies. As flat Vipp1 assemblies transition into barrel‐ (7O3Y) or tube‐like (8QHV) structures, the N‐terminal helix α0 becomes critical for membrane interaction. In these higher order assemblies, helix α0 promotes membrane curvature and stabilizes Vipp1 binding to the curved membrane surface. The resulting membrane‐internalizing tubular assemblies may remain membrane‐bound, be released into the surrounding environment, induce vesicular structures or facilitate membrane fusion events.

Electrostatic interactions via the α1–3 region of monomeric *Synechocystis* PspA and/or Vipp1 likely initiate membrane contacts. Once anchored to the membrane surfaces via the α1–3 hairpin, Vipp1 monomers can laterally associate on the membrane, mirroring the assembly behavior of eukaryotic ESCRT‐III proteins. The spiral structures observed upon membrane adhesion of cyanobacterial Vipp1 on solid‐supported lipid bilayers potentially are an initial step in the formation of barrel and/or rod structures that tubulate membranes, as recently discussed (McCullough & Sundquist, 2025). Cooperative polymerization on membrane surfaces ultimately leads to the formation of higher order structures, barrels, and/or rods (Hudina et al., 2025; Junglas, Kartte, et al., 2025; Naskar et al., 2025). The assembly process is tightly connected with membrane deformation, with inward tubulation and encapsulation of membrane segments within the polymeric protein scaffold rods (Hudina et al., 2025; Junglas, Kartte, et al., 2025; Naskar et al., 2025). Therefore, Vipp1 and PspA appear to employ at least two functionally distinct membrane‐binding regions: the α1–3 hairpin facilitates the initial electrostatic recognition of negatively charged membrane regions, triggering polymerization on membrane surfaces, while helix α0 becomes functionally important at later stages, mainly inducing membrane curvature and mediating stable interactions with the curved surfaces of internalized membrane tubules within assembled rods or barrels (Figure 4).

Notably, while this sequential model successfully accounts for membrane internalization during the formation of PspA and Vipp1 barrels and rods, it contrasts in some details with observations made in eukaryotic systems, where ESCRT‐III filaments have also been reported to bind membranes on the outer surfaces (Azad et al., 2023). In fact, ESCRT‐III proteins potentially engage membranes in multiple orientations.

In the case of Vipp1, an additional structural element, the C‐terminal extension forming helix α6, adds another layer of complexity. This region is predicted to form an amphipathic helix capable of inserting into lipid bilayers, indicating it could act as a third membrane‐interacting domain (Hennig et al., 2017). As helix α6 is localized on the outside of Vipp1 barrels or rods (Gupta et al., 2021; Junglas, Kartte, et al., 2025; Liu et al., 2021), this helix might enable Vipp1 to bind membranes on the external polymer surfaces. Thus, Vipp1 may combine conserved ESCRT‐III‐like activation and polymerization principles with unique structural alterations, enabling versatile and multivalent interactions with membranes during large‐scale remodeling events.

## THE FUNCTION OF CYANOBACTERIAL AND CHLOROPLAST MEMBRANE‐BOUND PspA AND VIPP1 ASSEMBLIES

Membrane‐bound PspA and Vipp1 form distinct polymeric structures linked to different functional states. In the case of Vipp1, these assemblies can be broadly classified into three categories: (i) flat structures, including spirals, sheets, carpets, and polygons; (ii) dome‐shaped structures; and (iii) tubular structures. For PspA, only the latter two are reported thus far.

It has been proposed that PspA and Vipp1 monomers, or smaller oligomeric assemblies, sense perturbed membranes and bind to these regions (Darwin, 2005; Gates et al., 2022; McDonald et al., 2015; Siebenaller et al., 2020). Two possible mechanisms have been suggested for the formation of flat assemblies. Either preformed rings are recruited to the membrane surface and subsequently disassemble to form extended carpet‐like structures (Junglas et al., 2020). In fact, membrane surface binding of preformed Vipp1 barrels has been shown (Hennig et al., 2015). Alternatively, monomers or small oligomers may assemble directly at damaged membrane sites into spirals, sheets, or polygonal lattices (Junglas, Kartte, et al., 2025; Naskar et al., 2025; Pan et al., 2025). The resulting spirals and carpets likely function as physical barriers or scaffolds that stabilize the membrane (Naskar et al., 2025). This mechanism could explain how PspA and Vipp1 prevent proton leakage (Junglas et al., 2020; Kleerebezem et al., 1996), as *E. coli* PspA in particular is known to maintain the proton motive force *in vivo* (Joly et al., 2010; Kleerebezem et al., 1996; Kobayashi et al., 2007). The idea of Vipp1 having a similar membrane‐stabilizing function is in line with the observation that both *Synechocystis* and *Arabidopsis* Vipp1 can substitute for PspA in a Δ*pspA E. coli* strain (DeLisa et al., 2004; Zhang et al., 2012). Consistently, *in vivo* experiments in cyanobacteria have shown that Vipp1 forms assemblies at TMs under stress conditions (Bryan et al., 2014; Quarta et al., 2026; Zhang et al., 2016) and that structures potentially corresponding to flat Vipp1 assemblies localize to stressed thylakoid regions (Gutu et al., 2018). While membrane binding of Vipp1 may involve disassembly of preformed barrel structure (Junglas et al., 2020), it currently cannot be distinguished whether disassembly occurs in solution, prior to membrane binding of monomeric Vipp1, or, alternatively, the preformed barrels bind to membranes where they disassemble to form carpets on membranes.

An alternative membrane repair mechanism involves the recruitment or formation of Vipp1 ring structures at damaged membrane sites, which may seal membrane lesions through budding and lipid leaflet constriction (Junglas et al., 2021; Liu et al., 2021; Naskar et al., 2025). These ring‐shaped assemblies could enclose lipids and thereby seal the damaged region via membrane shedding, as observed in eukaryotes during the plasma membrane repair process mediated by the ESCRT system (Jimenez et al., 2014). Such a process would require a membrane fission activity, which has recently been demonstrated *in vitro* for both *Syn*PspA and *Syn*Vipp1. *Syn*PspA remodels membranes *in vitro*, promoting both vesicle growth and tubular extensions as well as membrane fission events (Junglas et al., 2021). Similarly, *Syn*Vipp1 induces budding and forms coated vesicles *in vitro* (Junglas, Kartte, et al., 2025) (Figures 2a and 4).

Tube formation in the presence of lipids has been reported for both PspA and Vipp1, with lipids bound on the inner surface of the tubular structures (Hudina et al., 2025; Junglas et al., 2021; Junglas, Hudina, et al., 2025; Junglas, Kartte, et al., 2025; Theis et al., 2019). Furthermore, *in situ* experiments in *Chlamydomonas* have demonstrated that Vipp1 coats membrane tubules connecting the TM with the chloroplast inner envelope membrane (Gupta et al., 2021). Both PspA and Vipp1 show high structural plasticity within their tubular assemblies (Junglas, Hudina, et al., 2025; Junglas, Kartte, et al., 2025), and their ability to engulf lipids appears to depend on helix α0 (Hudina et al., 2025; Junglas, Kartte, et al., 2025). Helix α0 is proposed to interact with the membrane, induce local curvature, and thereby facilitate lipid tubulation (Hudina et al., 2025). For membrane‐attached PspA tubules, the release of vesicular structures from tubule tips has been experimentally observed (Hudina et al., 2025), consistent with the vesicle‐inducing activity that has been proposed for Vipp1 decades ago (Kroll et al., 2001). Furthermore, both PspA and Vipp1 exhibit a fusogenic activity and a membrane tubulation capacity (Hennig et al., 2015; Junglas et al., 2021; Thurotte & Schneider, 2019). Combined with the observation of Vipp1‐coated tubules connecting the TM to the chloroplast inner envelope membrane, this supports a functional role for these proteins in facilitating a direct lipid exchange, potentially via membrane‐bridging tubules, thereby contributing to intermembrane lipid transport (Figure 4).

It seems likely that flat Vipp1 structures can interconvert to curved barrel or tubular structures on membranes, as has been reported for other ESCRT‐III proteins (Pfitzner et al., 2021). Vipp1 spiral assemblies form ring structures at their centers, which extend outward and detach from the parent filament (Naskar et al., 2025). These rings resemble those reported to induce membrane budding (Naskar et al., 2025), suggesting that they either mediate membrane budding directly or represent an intermediate stage in the transition from flat Vipp1 assemblies to tubular structures. The latter could remain membrane‐attached or be released as protein‐coated membrane tubules (Junglas, Kartte, et al., 2025) (Figure 4).

The precise mechanisms underlying the formation and functional transitions of these distinct membrane‐bound assemblies remain unclear. Elucidating these processes will be essential for understanding TM biogenesis and repair, as well as how these functions are regulated through interactions with other proteins.

## PROTEIN‐MEDIATED MEMBRANE INTERACTION OF VIPP1

**Figure 5:**
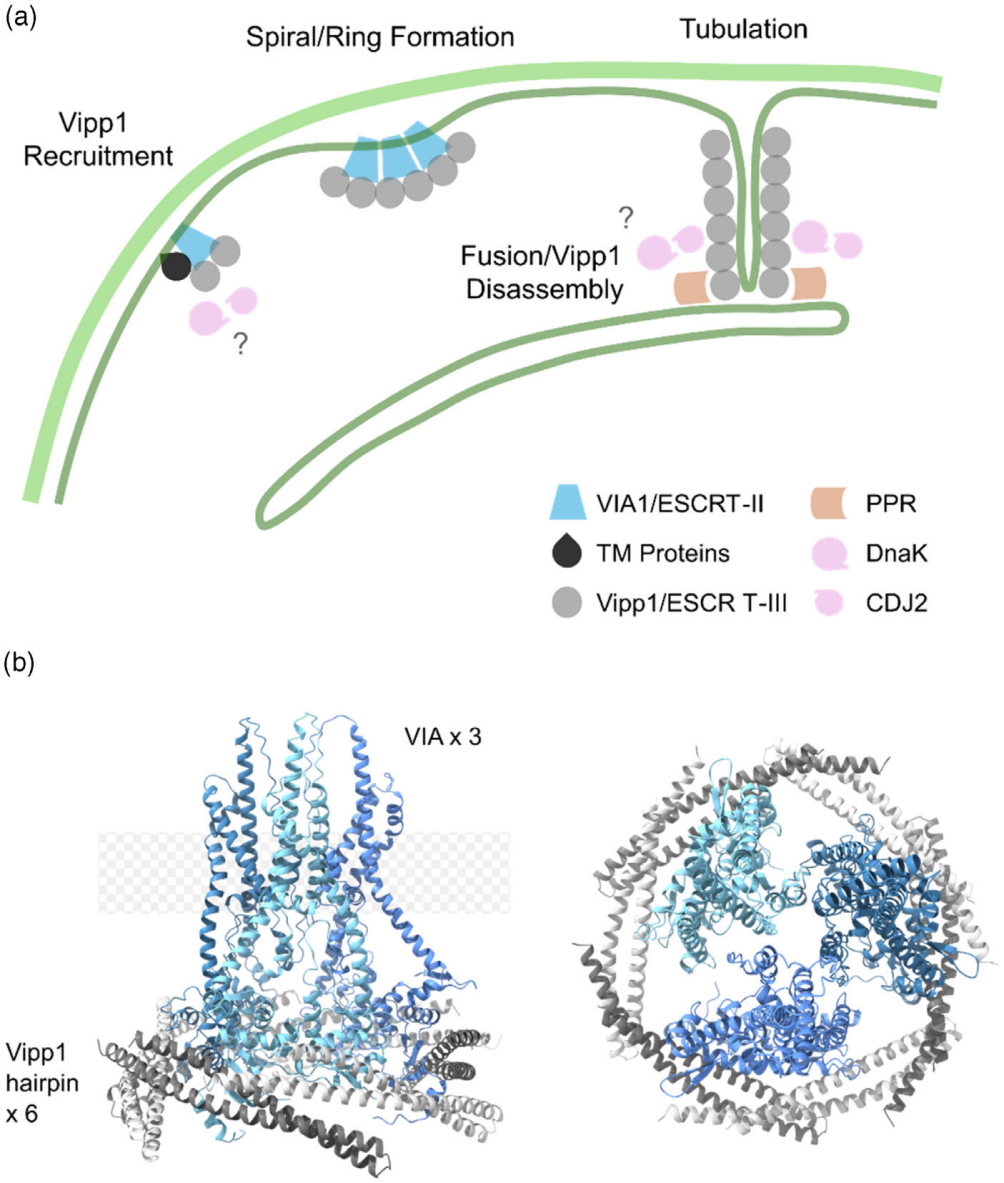
Protein interaction partners and protein‐mediated membrane interaction of Vipp1. (a) Model illustrating the putative roles of Vipp1 and associated proteins in plasma membrane remodeling, leading to invagination for TM biogenesis. TM proteins: small transmembrane proteins. (b) AlphaFold‐predicted structural model showing the interaction between three molecules of *Synechocystis* VIA1 (blue) and six molecules of Vipp1 (gray). In this AlphaFold‐predicted structural model, only α 1–2 hairpin motif of Vipp1 was used as input.

In chloroplasts and cyanobacteria, except for the ESCRT‐IIIs Vipp1/PspA, no direct homologs of other eukaryotic ESCRT proteins are present. Early attempts to identify Vipp1‐interacting proteins revealed two main classes of protein chaperones, the 70 kDa *heat shock proteins* (Hsp70, DnaK) and the J‐domain protein CDJ2, a chloroplast homolog of DnaJ (Bryan et al., 2014; Gao et al., 2015; Heide et al., 2009; Kreis et al., 2023; Liu et al., 2005; Liu et al., 2007; Yilmazer et al., 2025). These chaperones appear to be involved in the homeostasis and disassembly of Vipp1 polymers in chloroplasts (Li et al., 2025; Liu et al., 2007). More recently, several previously uncharacterized Vipp1‐interacting proteins have been identified in the chloroplasts of *Chlamydomonas* and *Arabidopsis* via proximity labeling‐mass spectrometry (PL‐MS) and immunoprecipitation‐mass spectrometry approaches (IP‐MS) (Kreis et al., 2023; Yilmazer et al., 2025) (Figure 5a).

Two proteins, evolutionarily conserved in all photosynthetic organisms, namely *Vipp1‐associated 1* (VIA1)/VPL3/CPLD50/Slr1603 and *fluctuating light acclimation protein 1* (FLAP1)/CPLD42/Slr0404, emerged as prominent Vipp1‐interacting partners. Additionally, several small transmembrane proteins (VPL4, VPL5, and At4g13220) and a member of the *pentatricopeptide repeat* (PPR) superfamily of proteins (At1g64430) were highly enriched in Vipp1‐intercatomes. These candidates are all membrane‐integral proteins comprising transmembrane helices anchoring them either in the chloroplast inner envelope or the TMs. Importantly, except for the small transmembrane proteins, homologs of the proteins identified in *Arabidopsis* and *Chlamydomonas* chloroplasts are also encoded in cyanobacteria, indicating a common origin.

These newly identified proteins show no overall sequence similarity to eukaryotic ESCRT proteins. However, two of them, VIA1 and PPR, contain characteristic ESCRT domains. VIA1 harbors two ESCRT‐II‐like winged‐helix domains, and PPR possesses an ESCRT‐III‐like α1/2 hairpin domain (Yilmazer et al., 2025). Similarly, an α1/2 hairpin‐containing protein, named *Vipp1 proximity labeling 2* (VPL2), has been detected in the *Chlamydomonas* Vipp1 proxeome; however, direct homologs in land plants and/or cyanobacteria have not been identified thus far (Kreis et al., 2023; Ostermeier et al., 2024). The PPR protein appears to be evolutionarily conserved in photosynthetic organisms. The algal homolog of PPR has about the same size as the *Arabidopsis* protein, whereas the cyanobacterial homolog lacks the N‐terminal region and retains only the hairpin domain and the transmembrane helices. Of note, not all cyanobacterial genomes code for a homologous protein (including *Synechocystis*), suggesting that the protein underwent gradual evolutionary refinement and reached its present form only in photosynthetic eukaryotes. The precise role of this PPR protein in membrane interaction of Vipp1 remains to be established.

In addition to Vipp1/PspA, the existence of associated ESCRT‐like proteins in both chloroplasts and cyanobacteria is intriguing and points toward a similar yet distinctively evolved membrane‐remodeling system. Although the molecular functions of these proteins in relation to PspA/Vipp1's recruitment to membranes remain uncharacterized thus far, roles analogous to that of auxiliary ESCRT proteins operating in the eukaryotic cytoplasm can be assumed. These roles could involve defining the sites of ESCRT‐III recruitment on chloroplast and cyanobacterial membranes or shaping ESCRT‐III filament topology on the membranes.

The ESCRT‐II‐like protein VIA1 is predicted to contain two winged‐helix domains (WHDs) arranged in tandem. Recent findings show that Vipp1 interacts with the WHDs of VIA1 via helix α1 (Yilmazer et al., 2025). Although this interaction interface closely resembles that of ESCRT‐II and ESCRT‐III proteins, VIA1 remains distinct from the canonical eukaryotic ESCRT‐II proteins. In eukaryotes, ESCRT‐II proteins typically consist of two tandem WHDs and form hetero‐tetrametric complexes. Some classical ESCRT‐II proteins, such as Vps36, harbor an additional GLUE‐like phospholipid‐binding domain that anchors the complex to membranes (Teo et al., 2006). The ESCRT‐II complex, consisting of four subunits, assembles into a characteristic Y‐shaped structure where the arms, exposing the winged‐helix domains, interact with ESCRT‐III subunits to promote filament formation (Henne et al., 2012; Im et al., 2009; Teis et al., 2010). In the case of VIA1, three membrane‐spanning helices appear to anchor the protein within the chloroplast inner envelope and cyanobacterial cytoplasmic membrane, respectively (Baers et al., 2019; Simm et al., 2013). Moreover, VIA1 appears to be the only ESCRT‐II–like winged‐helix domain‐containing protein in chloroplasts, suggesting the formation of a homo‐oligomeric ESCRT‐II‐like complex in the membrane. AlphaFold predictions of the *Synechocystis* Vipp1–VIA1 interactions support this assumption, yielding the highest scored structure with three *Syn*VIA1 (Slr1603) subunits anchored in the membrane that can bind α1/2 hairpin domains of six Vipp1 monomers (Figure 5b). In this arrangement, the Vipp1 monomers are positioned in a way that allows oligomerization via conserved inter‐monomer contacts. The predicted model suggests a role for VIA1 in initial membrane recruitment of Vipp1.

In cyanobacteria and chloroplasts, Vipp1‐mediated membrane remodeling is mainly associated with TM biogenesis and repair (Heidrich et al., 2017; Ostermeier et al., 2024; Zhang & Sakamoto, 2015). It is long believed and later supported by ultrastructure and lipidomic studies that the envelope membranes provide essential lipids for the biogenesis of TMs (reviewed elsewhere by (Mechela et al., 2019)). In line with this, loss of *via1* expression in *Arabidopsis* mutants resulted in reduced TM volumes and accumulation of numerous vesiculo‐tubular structures in the chloroplasts of newly emerging leaves (Yilmazer et al., 2025). This suggests that VIA1 is of special importance in the early stages of chloroplast development, where transition from proplastids to mature chloroplasts takes place and requires *de novo* TM biogenesis. Here, VIA1 potentially defines the sites of membrane invagination at the inner envelope of chloroplast via recruiting Vipp1 monomers, enhancing the pace of membrane remodeling and TM biogenesis. The suggested role is analogous to that of eukaryotic ESCRT‐II proteins. Still, it remains unclear whether VIA1 acts alone or alongside other Vipp1‐associated proteins at the chloroplast inner envelope membrane. The latter appears plausible considering the subtle effects of VIA1 absence on plant growth and development. VIA1 is evolutionarily conserved and functionally interchangeable between *Arabidopsis* and algae (Yilmazer et al., 2025). Given its high structural and sequence conservation, VIA1 may play a similar role in cyanobacteria, potentially assisting in ESCRT‐III recruitment to the plasma membrane. Yet, cyanobacteria appear to generate new TMs in the space between the plasma membrane and pre‐existing TMs, without a direct physical connection between the two (Huokko et al., 2021), and the suggested VIA1 function at the chloroplast inner envelope membrane (and cyanobacteria cytoplasmic membrane) does not explain Vipp1 recruitment to TMs.

The emergence of new Vipp1‐interacting partners in different model systems points to a distinct cyanobacterial/chloroplast ESCRT system bearing striking similarities to established ESCRT pathways found in the eukaryotic cytoplasm. As the influence of these *in vivo* identified factors on membrane interactions of cyanobacterial and chloroplast ESCRT‐III proteins is increasingly elucidated, we expect a more detailed understanding of the complexities underlying the ESCRT‐like membrane remodeling system in chloroplasts and cyanobacteria in the near future.

## CONFLICT OF INTEREST

The authors declare no conflict of interest.

## Data Availability

Data sharing is not applicable to this article as no datasets were generated or analyzed during the current study.
